# Understanding the lived experiences of medical learners in a narrative medicine course: a phenomenological study

**DOI:** 10.1186/s12909-021-02741-5

**Published:** 2021-06-05

**Authors:** Kuo-Chen Liao, Chang-Hsuan Peng, Linda Snell, Xihui Wang, Chien-Da Huang, Alenoush Saroyan

**Affiliations:** 1 Chang Gung Medical Education Research Center, Chang Gung Memorial Hospital, Taoyuan, Taiwan; 2 Division of General Internal Medicine and Geriatrics, Department of Internal Medicine, Chang Gung Memorial Hospital, 5 Fu-Shin Street, Taoyuan, 333 Taiwan; 3 College of Medicine, Chang Gung University, Taoyuan, Taiwan; 4 Institute of Health Sciences Education and Faculty of Medicine, McGill University, Montreal, Quebec Canada; 5 Department of Educational and Counselling Psychology, Faculty of Education, McGill University, Montreal, Quebec Canada; 6 Division of Chest and Thoracic Medicine, Department of Internal Medicine, Chang Gung Memorial Hospital, Taoyuan, Taiwan

**Keywords:** Narrative, Narrative medicine, Phenomenology reflection, Reflective learning, Transcendental phenomenology

## Abstract

**Background:**

Reflection and various approaches to foster reflection have been regarded as an indispensable element in enhancing professional practice across different disciplines. With its inherent potential to engage learners in reflection and improvement, narrative medicine has been adopted in various settings. However, the relevance and effectiveness of reflection remains underexplored in the context of narrative medicine, specifically in regard to the concern about variability of learner acceptance and the way learners really make sense of these reflective activities. This study aimed to explore what medical learners experience through narrative medicine and the meanings they ascribe to the phenomenon of this narrative-based learning.

**Methods:**

Using a transcendental phenomenology approach, twenty medical learners were interviewed about their lived experiences of taking a narrative medicine course during their internal medicine clerkship rotation. Moustakas’ phenomenological analysis procedures were applied to review the interview data.

**Results:**

Six themes were identified: feeling hesitation, seeking guidance, shifting roles in narratives, questioning relationships, experiencing transformation, and requesting a safe learning environment. These themes shaped the essence of the phenomenon and illustrated what and how medical learners set out on a reflective journey in narrative medicine. These findings elucidate fundamental elements for educators to consider how narrative approaches can be effectively used to engage learners in reflective learning and practice.

**Conclusion:**

Adopting Moustakas’ transcendental phenomenology approach, a better understanding about the lived experiences of medical learners regarding learning in narrative medicine was identified. Learner hesitancy should be tackled with care by educators so as to support learners with strategies that address guidance, relationship, and learning environment. In so doing, medical learners can be facilitated to develop reflective capabilities for professional and personal growth.

## Introduction

Reflection has been regarded as an indispensable element in professional education across disciplines [1–4]. The growing body of literature on reflection and strategies to foster it in medical education collectively provide a critical appraisal of interventions to better develop medical learners to become reflective practitioners [5–8]. Amongst these approaches, narrative medicine has drawn significant attention based on its promises for enhancing self-awareness and building affiliation with patients, colleagues and communities of practice [9, 10]. Not surprisingly, reflection stands out as one of the major themes when considering what learners take away from narrative approaches [11–14]. Researchers have not only expressed concern about formulaic approaches intended to engender reflection but have argued for redirecting attention about reflection to its theoretical orientation and keeping mindful of the challenges of applying reflection in different professional contexts [15, 16]. The variability of learner acceptance of reflection and reflective pedagogies have also been addressed as adding to the complexity of the phenomenon [17]. These concerns should alert educators to pay close attention to learner perspectives, especially on what meanings they ascribe to their lived experiences with narrative medicine so as to develop strategies to better engage learners in similar educational curricula that foster their reflective capabilities.

To contextualize the construct of reflection, we refer to Dewey’s [18] definition articulated in terms of “active, persistent and careful consideration of any belief or supposed form of knowledge in the light of the grounds that support it and the further conclusion to which it tends” (p. 6). By engaging in purposeful intellectual activities such as thinking back on events that have taken place in their professional practice, learners can better frame murky, complex problems [19–21]. Given previous researchers’ endeavors to conceptualize an iterative process of reflection and delineate vertical levels of reflective thinking, reflection has been positioned at the core of learning from experiences and practices [20–26]. In Mezirow’s transformative learning theory [24], learning is defined as ‘the process of making a new or revised interpretation of the meaning of an experience, which guides subsequent understanding, appreciation, and action’ (p. 1). The critical review of presuppositions and validation of premises consequently leads to transformations of learners’ beliefs, attitudes or emotional reactions. This act of critically reflecting on evidence, reasons, arguments, and alternative perspectives is central to adult learning theory as it is through this process that learners are able to make judgments, exercise decision-making, and develop the ability to adapt to changing conditions [24].

In medical contexts, deliberate, reflective practice has been argued to be an integral part of achieving and maintaining medical expertise and the process by which practitioners can generate adaptive strategies to address new situations [27, 28]. Medical educators have diligently integrated reflection into a variety of medical curricula with the expectation of improved clinical performance [11, 29–32], critical thinking skills and clinical judgment [33], professionalism and humanism [34], communication skills and empathy [14, 35], and diagnostic competence [36]. In their seminal systematic review, Mann et al. [5] provided recommendations on how reflection can be taught effectively. Moreover, they pointed to the necessity of further investigations of best practices that can achieve the goal of training reflective practitioners [5]. Despite these recommendations, researchers have striven to explicate theoretical models of reflection and curricular designs that can better foster reflection across the continuum of medical education [25, 26, 37]. Concurrently, studies that have aimed to explore the effectiveness of the application of strategies for developing reflective capacities have provided promising evidence and innovative ideas [14, 38–41]. Among these approaches, narrative medicine, though being adopted or adapted for use in different contexts, has gained a growing acceptance to meet the need of cultivating reflective practice in medical education [35, 42].

Narrative medicine, advocated by Charon [9] since the early period of twenty-first century, is ‘medicine practiced with narrative competence to recognize, interpret and be moved to action by the predicaments of others’ (p. 83). With the development of narrative competence and narrative-based practice, health professionals manage to delve into the stories of patient illnesses they have encountered in clinical workplaces through paying attention to, representing and connecting with others and self [9]. Healthcare professionals may have a heightened sensitivity to relational interactions in medicine that go beyond traditional disease-framed practices [43]. Since narratives embody intricate human experiences, writing narratives allows learners to establish a link with knowledge, contexts and experiences of practice, and enables them to ‘understand the meaning and significance of stories through cognitive, symbolic, and affective means’ (p. 1898) [35]. Incorporating prompted narrative writing into clinical learning curricula can lead to reflection, promote self-awareness, and provide an emotional outlet for learners [44]. Wald et al. [14] described a structured curriculum with interactive narrative writing assignments in which medical students received faculty guided feedback to promote reflection. To this end, a narrative approach may create pedagogical spaces for professional development by facilitating reflection, and therefore enhance learning from clinical encounters with patients [45].

Studies have shown that the inclusion of narrative medicine supports individual, interpersonal, perceptual and expressive capacities [13], enhances the richness of experiences from doctor-patient interaction [46], and brings about a deeper appreciation of the human side of medicine for medical learners [47]. Reflection has been identified as an important vehicle for and/or an outcome of these similar narrative medicine curricula [11, 42, 48, 49]. In order to achieve the goal of fostering reflection by using narrative approaches, it is pertinent for educators to understand learner perceptions and experiences. With better understanding of what meaning learners ascribe to reflective learning in narrative medicine, educators will be better equipped to employ educational strategies for future development. Literature on perceptions of reflective learning in the context of narrative medicine is limited. In order to address the gap in the literature, this study aimed to explore the lived experiences of medical learners in a narrative medicine course. Two research questions were asked:

1. What are medical learners’ lived experiences in a narrative medicine course?
2. How do medical learners experience learning with narrative medicine?

### Research design and methodology

Phenomenological research aims to explore the essential, invariant structure or the central underlining meaning of individuals’ lived experiences. By determining what an experience means as it is subjectively lived by individuals who have had the experience, a comprehensive description of it can be provided [50, 51]. Several schools of phenomenological research have drawn on German philosophy to seek to understand what it means to be in the world or human experience as it is lived [51, 52]. In Husserl’s transcendental phenomenology, the researcher’s goal is to search for an understanding of the meaning of the participants’ experiences based on the stories being told from the participants’ voices, and further move from these experiences to describe the essence of the phenomenon [53]. This descriptive approach requires the researcher to set aside previous understandings or presuppositions, a process of *epoche* or *bracketing*, and achieve the state of *transcendental I*, a state of being required for fresh perception of the participants’ experiences to depict the essence of the phenomenon [54]. In comparison, hermeneutic phenomenology, originated from Heidegger’s interpretive phenomenological tradition, requires the researcher to focus on the relationship between participants and their *lifeworld* and develop a reflective interpretation of participant accounts to achieve a meaningful understanding of a phenomenon [55].

Drawing on Husserl’s transcendental phenomenology, Moustakas illustrated a systematic approach to analyze phenomenological data with a focus on searching for a composite description of a phenomenon that captures the meanings and the essence of experiences [51]. In line with the purpose of this study to understand the nature of learner lived experiences with narrative medicine, Moustakas’ transcendental phenomenology [51] was chosen to delineate the essence of how medical learners interpret, process and experience learning in narrative medicine.

### Research context and the narrative medicine course

A narrative medicine course was incorporated into a three-month internal medicine clerkship for 5th year medical students at a tertiary hospital in northern Taiwan. In contrast to previous studies on narrative medicine, this course modified the common three-step model of read-reflect-respond [42] by structuring in three parts: (1) an introductory lecture on narrative medicine at the beginning of the internal medicine clerkship; (2) an independent narrative writing session in which each learner prepares a personal narrative based on a chosen clinical experience during the clerkship; (3) an educator-facilitated small group discussion in which learners share personal narratives near the end of the rotation. The educators participating in this course are attending physicians of internal medicine with more than 2 years of experience in facilitating small group discussion in the narrative medicine course. In this course, each learner is required to compose one narrative writing assignment in which he or she writes about a patient’s story or a clinical experience then embellishes it with personal written reflections. These narrative writing assignments, designed to be used as prompts for reflection and discussion, are then shared in a small group comprising five to eight medical learners.

### Epoche (bracketing) and researcher reflexivity

Epoche is defined as “the elimination of suppositions and raising knowledge above every possible doubt” (p. 26) [51]. Based on Husserl’s concept of epoche (or bracketing), Moustakas emphasizes that researchers should strive to set aside their previous experiences so that they can perceive the phenomenon under examination freshly, as if for the first time [50]. Consistent with the first part on Moustakas’ systematic approach, in order to put aside previous bias and presumptions, the primary researcher (KCL) deliberately reflected on personal experiences of participating in the narrative medicine course, including small group discussions and feedback on students’ written or verbal reflections.

The primary researcher (KCL) began his involvement in the narrative medicine course as a small group facilitator in 2010. As he read and listened to learner’s accounts of their clerkship experiences in narrative writings, he began to appreciate the reflections they shared and provided feedback and encouragement to learners. These moments brought back a vivid image into his mind about his own learning experiences. Thinking back, he saw a hesitant, timid medical student wearing a dignified white coat, shuffling back and forth in the hospital wards, acting like a professional, striving to hone clinical skills, and showing so-called ‘empathy’ or ‘compassion’ in a professional manner. As he moved along his trajectory of becoming a doctor, he witnessed moments of pain and suffering among patients and their family members. Through his engagement in the narrative medicine course, the primary researcher’s belief of delivering a humanistic practice was strengthened, and furthermore, his identity as a physician was molded firmly by taking into account patients’ life stories in his clinical decision-making and patient care. When the primary researcher received graduate training as a Master’s student in health professions education, his belief about reflection and its’ importance in professional development became even more steadfast. These experiences collectively added interest to his exploration about the meaning of reflection perceived by medical learners in narrative medicine.

The primary researcher presumed that the activities as described in the narrative medicine course should be facilitative for learners to reflect on their experiences. However, upon reflecting on his experiences of employing this educational strategy, he was doubtful about whether students would intuitively know what narrative medicine was or they would be able to recount not just an incident but also be able to incorporate the emotions, feelings and thoughts that surrounded the event. Such feeling of uncertainty in regard to learners’ experiences of preparing narrative writing assignments and their responses in the small group discussion prompted primary researcher’s curiosity about the phenomenon of learner reflective learning in narrative medicine which typifies the first stages of Moustakas’ heuristic methodology [56].

These processes of recalling previous experiences with narrative medicine and reflection were repeated until the primary researcher felt he had achieved a state of receptiveness to concentrate fully on the participants’ experiences in explicating the meaning of their engagement in the narrative medicine course. Besides, the primary researcher had taken time out from his clinical work in Taiwan and secured an opportunity to develop and conduct this study during his Master’s program. Given these opportunities, and deliberately bracketing previous experiences in narrative medicine, the primary researcher developed sensitivity of being aware of his preconceptions and their possible influence on conducting this study.

### Research team

The research team comprised researchers from different backgrounds with various areas of expertise. CDH is a chest physician and clinician-educator, who is the narrative medicine course designer. He shared the belief that narrative medicine would be a beneficial venue to introduce humanistic, holistic patient care for medical learners and would be an enabler to heighten empathy in health professionals. LS and AS were the primary researcher’s supervisors for his Master’s program in health professions education. They have extensive experiences and research achievements in learning science, educational psychology and health professions scholarships. XHW has a Ph.D. in educational psychology and is a trained qualitative researcher. CHP is a trained qualitative researcher with a background in educational and counseling psychology and works as a research assistant. In addition to the stage of *epoche* described above, the research team collectively reflected on their experiences with narrative medicine, shared their thoughts and beliefs of reflection and related narrative approaches in professional education, and iteratively discussed and interpreted the research findings in regular meetings.

### Participants

Medical learners who had had experience with the narrative medicine course were considered as potential participants. A purposive sampling was employed to recruit 20 participants who had experienced the phenomenon of the study focus. This approach complied with recommendations on study sampling in previous phenomenological research and qualitative studies [50]. Invitation letters were distributed across medical learners’ public social media, such as class blogs. Those who were interested in participation voluntarily contacted KCL and CSP were informed of the study purpose and were invited to sign a written consent form for participation. The study received research ethics approval from both the Institutional Review Board at Chang Gung Memorial Hospital in Taiwan and the Research Ethics Board at McGill University in Canada.

### Data collection

This study was conducted from February 2015 to September 2016. Each enrolled participant provided demographic data via a written questionnaire and then took part in a one-on-one in-depth interview. The demographic information included age, gender, year of medical training at the time they participated in this study. In keeping with Moustakas’ framework of phenomenology, the interviews focused on collecting participants’ depictions of their lived experiences to understand the central phenomenon in this study [51]. The interview guide was informed by previous literature on narrative medicine and reflection, and developed based on the discussions within the research team (see Appendix 1). The interviews were conducted in Chinese for the participants to freely elucidate and share their experiences and each individual interview lasted between 45 to 60 min. Two researchers, KCL and CSP, conducted the interviews by using the interview guide to maintain interviewing consistency. For participants’ convenience, KCL interviewed seven participants via Skype and CHP conducted face-to-face interviews with the remaining thirteen participants. All interviews were audio-recorded, transcribed verbatim and anonymized before analysis.

### Data analysis

In accordance with the principles of a phenomenological research, coding was initially open-ended and inductive to allow the researchers to pay careful attention to participants’ accounts [50, 57]. Two Chinese-speaking researchers (XHW and KCL) read seven anonymized transcripts several times to gain familiarity with the data and to immerse themselves in the lived experiences of the participants [56]. An open coding procedure [58] was applied and a list of coding categories was finalized through an iterative process which involved discussion between two independent coders to resolve any disagreements. The coding scheme was then reviewed by the research team, and then achieved consensus through iterative discussion. Following that, a third researcher, CSP, recoded all the data accordingly using the agreed-upon categories and utilized MAXQDA 11software (2012,VERBI GmbH, Berlin, Germany) for completing coding and data management.

Moustakas’ phenomenological analysis was then employed with major processes summarized in Appendix 2 [51]. The primary researcher (KCL) and CSP identified a list of significant statements about how participants experienced the phenomenon of interest, and grouped the statements into meaning units, or themes. A subset of the interview transcriptions and significant statements were translated into English by KCL to allow the non-Chinese speaking co-researchers to engage in data analysis. The research team reviewed and refined the results through team discussions and reached consensus. A composite construct of the meanings drawn on participants’ lived experiences was presented. This yielded a description of ‘what’ the participants’ experienced concerning the phenomenon, ‘how’ their experiences happened, and an integrated synthesis about the essence of the phenomenon [50].

### Trustworthiness and rigor

Several validation strategies were adopted to enhance the trustworthiness and rigor of this study [50]. First we performed a member check with the participants. This involved returning a synthesis of each participant’s data, and a draft of analyses and interpretations back to participants asking that they read to judge the accuracy of what was captured as their perspective. This process increased credibility of the results. Second, the disciplinary diversity of the researchers and their scrutiny of the data treatment procedure and application provided another level of trustworthiness. Third, the primary researcher (KCL) has a background as a facilitator of the narrative small group discussion. He took code notes and memos during the data analysis process to ensure that as the researcher, he examined his own assumptions in the context of the data and focused attentively and reflectively on the participants’ perspectives on their lived experiences of the phenomenon.

## Results

Twenty medical learners participated in this study and their demographic data are presented in Table 1. Two-hundred and fifty five significant statements were identified in which participants elaborated their experiences with narrative medicine (see Appendix 3 for selected examples). In this process of *horizonalization* (see Appendix 2 for details), we pondered deliberately on participant utterances and got to understand the textural qualities of the phenomenon they experienced. According to Moustakas’ procedures of phenomenological analysis, these statements were then grouped into clusters of themes or meaning units, as presented in Table 2.

**Table 1:** Demographic characteristics of the participants

Characteristic	Value
*Age in years, no. (%)*	
< 25	15 (75%)
25–35	5 (25%)
*Gender, no. (%)*	
Male	7 (35%)
Female	13 (65%)
*Department, no. (%)*	
Medicine	9 (45%)
Chinese Medicine	11 (55%)
*Grade, no. (%)*	
5th Year MS^a^	4 (20%)
6th Year MS	9 (45%)
7th Year MS	6 (30%)
1st Year Resident	1 (5%)

^a^ Medical student

**Table 2:** The six main themes identified from the participants’ lived experiences in regard to learning in narrative medicine

Themes	Abbreviation
Feeling hesitation	Hesitancy
Shifting roles in narratives	Narrative shift
Experiencing transformation	Transformation
Seeking guidance	Guidance
Questioning relationships	Relationship
Requesting a safe learning environment	Learning environment

Participants’ elucidation of their experiences about learning in narrative medicine was captured in six themes: feeling hesitation, seeking guidance, shifting roles in narratives, questioning relationships, experiencing transformation, and requesting a safe learning environment. Each of these themes is elaborated below.

In addition, participant excerpts were provided to demonstrate the grounding of findings in the data. Each excerpt is followed by participant pseudonym, recruitment number, gender and level of training achieved at time of interview. Within the transcript excerpts the following conventions were used: the bracket () shows additional clarifying information, the semi-colons […] means omitted texts; and the ellipse (…) means a pause [59].

### Feeling hesitation

Participants showed hesitation in regard to engaging in the narrative medicine course because they were not sure about what to do and many of them were slow to act on writing narratives. Most participants perceived narrative medicine as a course similar to medical ethics or humanities. Upon asking about their experiences with the narrative medicine course, a majority of participants contended they had already taken many similar courses. One participant disapproved this repetition and stated,

Again, they are attempting to give us some high-sounding knowledge or distant, irrelevant concepts. And the content seems to be pretentious. Frankly speaking, I almost run out of interest to learn. (P2, F, MS5)

Participant hesitancy was underpinned by doubtful moments of the proclaimed benefits of humanistic courses, uncertainty about what the narratives were for, and even more fundamentally, how to write narratives. Some participants expressed a resistant attitude toward endeavoring to write narratives that conflicted with learning biomedicine in the clinical environment. As one participant expressed,

I don’t think just taking these kind of courses will be helpful to improve our communication skills or build a good doctor-patient relationship. (P6, F, MR1)

The humanistic features of narrative medicine or so-called ‘soft skills’ had apparently challenged participants’ assumptions regarding learning clinical medicine, particularly their presumed focus on acquisition of medical knowledge, diagnostic skills and biomedical competence as they stepped into clinical learning environments. One participant shared her thoughts as follows:

For me, what narrative medicine can provide is some modification of humanism. I strongly believe that most of the learners rarely spend time on reflection (or reflecting on their experiences). […] They are high achievers in biomedical performance and I contend that this should be the most important part of being a doctor. After all, we are not here to be trained as a social worker nor a counseling psychologist. What matters most are your treatments for patient aliments’ (P3, F, MS5).

### Seeking guidance

Since participants were requested to write and share personal narratives in the course, they perceived a need to gain more guidance at the outset regarding how to write personal narratives. One participant commented,

I felt that we were given a haphazard journey. We did not receive the needed guidance… I had no idea about how to write a narrative or even just to find a starting point. To be honest, I had doubted about this (how to write a narrative) for a while.’ (P1, F, MS7)

Most participants completed the assignments either by intuition, using their imagination, or through imitating what they saw as narrative exemplars posted on a recommended website. One participant explained how she achieved an understanding about preparing the assignment:

After attending to the introductory class, I spent a while to look over the posted narratives on the website in order to understand some details about how to prepare for this assignment. I had a sense that they were writing a fiction…a short story with comments on what they have learned. (P3, F, MS5)

Despite participants expressed uncertainty about this required task, they came up with personal interpretations in writing narratives and aimed at getting a good mark on assessment. One participant stated:

If the instructor want to achieve the goals of this course, it would be helpful for him to explain more details about how to write narrative assignments. Our imagination of narratives was to write a touching story and then you could get a high score for it. (P7, F, MS5)

### Shifting roles in narratives

By writing narratives, participants shifted between their roles and the protagonists in narratives so that they could experience the story in a sensitive, humanistic way.

To me, this is a kind of shift. It is this shift that makes you change to another mode, a sensitive and humanistic one. The stories are commonplace in the medical context. However, most of the time we tend to put our efforts on the scientific aspect of medicine. (P1, F, MS7).

This act of role-shifting enabled participants to reconnect what they had seen with how they had felt in clinical encounters. One participant described her experience of role-shifting and expressed her empathy by putting herself in someone else’s shoes:

If I was the patient’s mother, neither a physician nor a medical student, and I happened to know that this group of health professionals knew more truth (about my son’s illness) than me, I would be extremely, extremely upset…, and I would be even reluctant to accept my role as the patient’s mother.’ (P3, F, MS5).

Participants recalled some startling moments during clinical placements and their act of writing helped them to review their emotions provoked by these experiences. One participant stated her experience with a patient needed to receive endoscopy examination:

I wrote my story about accompanying a patient to receive an endoscopy examination because my clinical supervisor asked me to do so. When I witnessed the struggles a patient needed to go through and the discomfort he had to tolerate during the procedure, I came to a realization why most patients would be resistant to receiving an invasive procedure like this (endoscopy). (P11, F, MS6)

### Questioning relationships

In the narrative medicine course, participants were assigned randomly by the administrative staff to discussion groups facilitated by different educators. They expressed concern about the unfamiliarity in a small group activity, which led to a relationship without trust, impeded their sharing of thoughts and reflection, and consequently formed a barrier to effective participation. One participant recounted his experience in a small group discussion with unfamiliar, diverse group members:

The acquaintance level between the members was very important. […] If we did not know each other to some extent, how could I share my feelings from the bottom of my heart without hesitation? (P4, M, MS6).

As participants transitioned from medical schools into clinical learning environments, they were assigned to different learning or practice groups in various contexts. This cultural shift made participants feel anxious and even embarrassed upon being asked to share their narratives. One participant stated,

If there is someone in the group who you are not acquainted with, you will shy away from sharing your narrative. This issue (being in an unfamiliar discussion group) may embarrass people. (P15, F, MS6)

As a result, participants tended to give superficial comments and polite remarks. This kind of relationship-building had generated disbelief and low expectation for group discussion. Another participant expressed,

We were very anxious in a small group setting because the educator asked everyone to comment on another member’s narrative. For any reason, you would try not to give negative feedback. Hence, most comments fell into the similar (superficial) category. I did not expect too much for the discussion. (P1, F, MS7)

In response to a disappointing relationship which exuded lack of mutual trust, one participant voiced her experience:

This kind of group and discussion holds no promise for reflection. I don’t think you would express your own feelings or thoughts in front of a nearly unfamiliar educator and several unfamiliar colleagues. […] Such an unfamiliar group combination for this course would hardly result in any sparkles….It was, I would say, meaningless and superficial. (P3, F, MS5)

### Experiencing transformation

As participants engaged willingly in the narrative medicine course and set out on the learning journeys, they experienced transformation in various aspects, either in the aspects of cognition, affections, or behaviors. From the selection of topics to the process of writing, participants appreciated narratives as a vehicle for distilling emotions and heightening self-awareness and sensitivity. Writing narratives also provided an opportunity for participants to organize their thoughts and solidify their thinking in the complex clinical settings. Furthermore, participants experienced validation of their emotions by the educator or the group members when they shared their narratives in small group discussions. They expressed they were enabled to compose themselves, make sense of their experiences in unsettled moments, and generate practical strategies that could inform their future practice in similar situations. As one learner stated:

Reflection is, I think, just like you compose your emotions throughout a process. […]. Since you have thought of it, you would be more conscious of and confident about what you could tell your patient […]. I don’t want those tangible moments to become meaningless for me after I have practiced for a longer period of time. (P7, F, MS5)

Participants described their participation in the narrative medicine course as transformative. With immersion in writing narratives, participants contended that they could revisit their experiences in a humanistic way. As participants started to prepare their narratives, they were appreciative of the deep thinking and continual inquiry inspired by the act of reviewing, writing, and sharing in the narrative medicine course. One participant elaborated his experience as a process of making the unnoticed noticed:

After I put efforts in my writing and thought about my process of patient care for a while, I realized that there were so many details I formerly overlooked. But I should have noticed them […]. This kind of deep thinking was not likely to occur before you started to write your narrative. (P4, M, MS6)

When participants rediscovered more details after reviewing clinical experiences, their provoked emotions were consolidated and they managed to sort out potential solutions for future practice. One participant described how his personal repertoire was enriched in this course:

Initially, I felt a difficulty to transform an emotional moment into a readable story in my narrative […]. But when I try to sit down and write, I discovered that there were a lot of details emerged from my review of that particular event and it was invaluable for me to think again. […] I came up with a solution in my writing which was: maybe at that time I could have brought the patient to another quiet room and let the rushing pace slow down. (P2, F, MS5)

Through engaging in the reflective journey of narrative medicine, participants had the opportunity to rethink about what are the values of being a doctor with empathy and compassion: As one participant described,

For the first time, I would like to pay attention to the patient on daily visits, and want to accompany him and care about whether his condition did improve or not. This made me reflect on what the values are to be a doctor. It does not merely lie on the cure of his disease but heavily on how you care him as a person. (P20, F, MS5)

### Requesting a safe learning environment

Participants recounted the learning environment in which narrative medicine took place was key to shape their experiences, particularly about how they choose to share personal reflections in a small group discussion. Aspects mentioned by participants included the group size, member composition, scheduling issues, and physical spaces. For example, some participants shared their narratives in a ward meeting room, and some exchanged their reflective thoughts at a fine restaurant which served food and drinks. As one participant stated,

The environment counts (for sharing our narratives). Some group held the discussion at a restaurant “The Rose Garden”, which was conducive for thinking and sharing. In contrast, our group members were crowded in a small meeting room. It was a tight squeeze and I just wanted to leave as soon as possible. (P12, F, MS6).

With regard to sharing authentic personal reflections, participants requested a sense of security to express their emotions, feelings, and even vulnerabilities in front of the group members and the educator. One participant shared her sensitive feelings and exposed personal weaknesses in a small group context and gained a sense of being inclusive among the peers:

We held the discussion in a café. It was a relaxed place and conducive for sharing […] After I shared my experience about my overwhelmed feeling and sense of incompetence upon facing a dying patient, the shared emotions resonated among group members I was quite relieved when I realized that other colleagues also experienced similar situations. (P2, F, MS5)

Participants also asked for modeling of written or verbal reflections in narratives by educators. Notably, they were enthusiastic about listening to educators’ experiences that echoed the issues highlighted in learners’ narratives. One participant stated,

Since the teachers were experienced clinicians, it would be better for us to read teachers’ narratives or reflections as a guide for us to prepare our own narrative assignments. (P14, F, MS6)

Once the climate is not supportive and the process is perceived as only a formality, the learning environment is detrimental to learners’ reflections provoked by writing or listening to narratives. One participant recounted her experience of a group discussion that showed the absence of a shared understanding about the goal of the activity:

The educator was not clear about the goal of the course. He just knew that he had to give a mark on each narrative writing assignment. We scuffled through our narratives one by one. This act, to certain extent, discouraged the writer and the audiences, and prevented us from engaging in an in-depth discussion and group interaction. (P17, F, MS7)

As well as the above six identified themes a description of ‘what’ was experienced (textural descriptions) and ‘how’ the phenomenon was experienced (structural descriptions) was provided.

### What did the participants experience in a narrative medicine course?

The participants described their respective journeys by using words such as ‘haphazard’, ‘uncertain’ or ‘doubtful’ and they had shown ‘hesitancy’ to take on the course. At the outset, participants were unable to foresee the proclaimed benefits of writing narratives and perceived the course as irrelevant to their learning of clinical medicine. Some participants even regarded the course as a ‘redundancy’ because they had taken similar preclinical medical humanities courses in early years of medical school. While most participants felt that they were being ‘coerced’ to write personal narratives, one participant still commended this course as ‘a promising trial’ to complement biomedical dominance in their clinical training. Although most participants did not have a writing habit to jot down notes about humanistic issues during their clinical placements, a receptive and positive attitude was found in one participant who had a habit of keeping a diary and showed an active interest in observing people in the workplace. Even with hesitancy, participants appreciated the act of writing individual narratives and deemed the reflective learning experience as ‘a rediscovery of self’ and ‘rethinking about who he wants to be’. Furthermore, participants gained a sense of security when dedicated educators led small group discussion in a safe and supportive learning environment.

### How did the participants experience the phenomenon of learning in narrative medicine?

As the participants reflected on their experiences, the timing of implementing this course played an important role. Despite the delivery of the history about narrative medicine and the evidence of its benefits in the introductory lecture, participants described it was rather a high sounding preaching lecture and irrelevant to themselves when they were in the very early stage of learning clinical medicine. Their focus of learning centered on the biomedical aspect of medicine with eagerness to mastering different kinds of clinical skills. This proposition impeded participants to develop a holistic, humanistic approach to looking at events from different angles without clear guidance, particularly when they encountered complexity of clinical medicine. Besides, participants had taken several mandatory courses on medical humanities in their undergraduate years. Some of them conceived narrative medicine as ‘an add-on task’ and even regarded it ‘disruptive’ to their learning in the clinical environments. Bearing the hesitancy, doubt and uncertainty in mind, participants expressed low expectation towards the small-group discussions and they tended to participate in the narrative activities in a perfunctory manner.

Participants sought guidance at the outset, either by individual inquiry effort or by listening to words from the mouth of peers. They were driven by the goal of getting a high grade by emulating the narrative exemplars to write what should be included in the content. Guidance experienced in the group discussion facilitated participants to seek role models from educators’ experiences when they encountered similar clinical situations and supported participant identity formation as becoming a physician. Educators’ commitment to narrative discussions strongly encouraged participants in reflection and facilitated their journeys with constructive feedback and mutual relationships. Instead, with insufficient efforts dedicated by educators hampered participant engagement in these designed reflective activities. However, time constraint and the educators’ workload in a busy clinical environment were reasons for ineffective experiences with the narrative medicine course and resulted in a mere formality conceived by the participants.

From the descriptions of our participants, the randomness of group assignment was deemed detrimental to trustful relationship-building and authentic sharing of feelings and emotions in narrative group discussions. In addition, when participants recounted their lived experiences about the physical space in which group discussion took place, a relaxing atmosphere conducive for reflection at an elegant restaurant stood in stark contrast to a narrow crowded space full of multiple disruptions, like the meeting room at a medical ward. These aforementioned contexts constitute the groundings connected with textural descriptions of what participants experienced in the narrative medicine course and, furthermore, explain how the participants make sense of these experiences with narrative medicine. A synthesis of the essence and meaning of the phenomenon is presented in Table 3.

**Table 3:** A synthesis of the essence and meaning of learners’ experiences with narrative medicine

Learning in narrative medicine begins with learner hesitancy in response to the course requirements, writing a personal narrative and then sharing it in a small group. Learners’ conceptions about narrative medicine, shaped by previous assumptions of and experiences with humanistic courses, influence their attitudes as they set out on this journey. Learners show substantial hesitation at the outset, either being in doubt about choosing what to write about or uncertain about the proclaimed benefits of narrative medicine, and even show resistance to participate in the course or engage in writing narratives. However, writing narratives creates a space for learners to reflect and serves as a vehicle for shifting roles in narratives. It encourages learners to put themselves in patients’ shoes, to look at things from different angles, and to raise sensitivity to patients’ needs in clinical practice as healthcare professionals. In doing so, learners may experience transformation in several aspects, such as heightened self-awareness, emotion regulation, perspective changes, and generation of action plans in response to various clinical circumstances. Learners become empowered along the journey by achieving better understandings about patients and enriching personal repertoires for dealing with similar situations in future practice. Notably, learner hesitancy is either alleviated by the guidance they seek, accompanied by the relationships built among the educators and group members, and supported in a safe learning environment, or it can be deepened at the next level whenever learners fail to gain guidance, question insecure relationships in a subversive learning climate along the journey.	

## Discussion

**Fig. 1:**
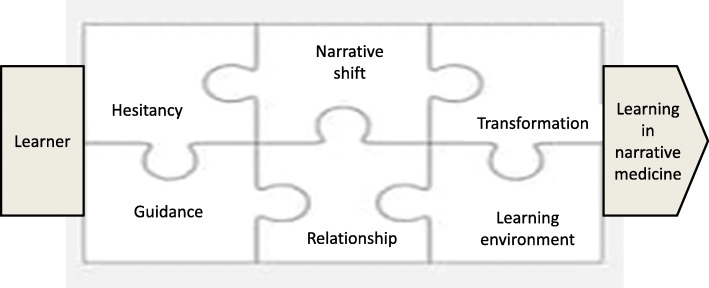
Model of the essence of learning in narrative medicine

By adoption of a transcendental phenomenology approach, this study was a first to deeply explore medical learners’ lived experiences with narrative medicine, particularly focusing on what and how they experienced the phenomenon of learning in narrative medicine. A model that depicts the essence of the phenomenon is presented in Fig. 1.

From the current study, the analysis indicated that learning in narrative medicine begins with participants’ hesitancy in response to facing the mandates of taking this course, writing a personal narrative and then sharing it in a small group. Feeling initial hesitation and pressure within what could be described as turmoil for participants at the outset could be analogous with anyone setting out on an unknown journey. However, participants’ engagement with writing narratives could be facilitative for them to make a narrative shift to complement what they have perceived as orthodox learning of clinical medicine. With this act, participants experienced role shifting in the process of preparing for, pondering about and writing down their reflections and finally sharing their narratives in small group discussion. Participants experienced transformation in cognitive, affective, and behavioral aspects and felt empowered with enriched personal repertoire of action plans to face difficulties and challenges in learning medicine. Furthermore, guidance, relationship, and learning environment constitute the prerequisites to support participants in reflective learning and practice. Gaining sufficient guidance sustains participants to hold fast their journeys so that they can engage in processes of writing and sharing narratives. The educator-learner or peer relationships not only validate learners’ feelings of being understood and comforted but also reciprocally nurture participants’ professional growth and identity formation. A safe learning environment invites participants to reflect on their clinical experiences and encourage personal sharing to sustain the influences of narrative competence both at individual and collective levels. In the results of this study, contextual factors that made these elements insufficient or posed negative effects on participants’ experiences would finally lead to an undesirable outcome in regard to adopting a narrative approach to facilitate reflection.

Learner hesitancy is a complex and context-specific state, which may stem from learners’ emotions of doubt, uncertainty, or resistance to medical humanities courses such as narrative medicine [60]. Since emotion plays an important role in learning, learners’ emotional responses to the designed learning activities are often the most influential factors affecting their engagement in these activities [15]. As Dewey [18] argued, one of the elements in reflective thinking is ‘a state of perplexity, hesitation, doubt’ (p. 9) and participant hesitancy brought about an act of search for solution to the problem involved in their lived experiences with narrative medicine. Our participants disclosed their attitudes toward narrative medicine and even some of them were confused or struggled with their presuppositions about learning reflective capabilities and writing narratives. Although literature on narrative medicine has described its legitimate position in medical education, few studies have discussed about such an ambiguous circumstance [17, 61, 62]. As indicated in our results, participants described their resistance related to previous experiences with taking prior medical humanities courses. This finding is consistent with Shaprio et al. [60] who described several areas of discontent about medical humanities form learners’ perspectives and why medical humanities usually engenders criticism or even contempt. Learners tend to embrace the ideas of ‘uncertainty’ and ‘resistance’ because of perceiving reluctance or lack of relevance [60, 63].. When learners transition into clinical learning environments, as the participants in this study did, they are likely to choose the prevailing mechanistic, dominant biomedicine mindset and decide what to learn in their daily activities. In regard to develop reflective capabilities by choosing a humanistic, narrative approach, feelings of hesitation may unsettle learners’ self-assurance in what they know and activate problematic conditions that are detrimental to reflective learning. These unsettling experiences may be harnessed by providing guidance from the pedagogical design, the relationship built with and role modelling of reflection by educators [14, 29]. Hence, it is critical for educators to be cognizant of and sensitive to learner hesitancy in educational curricula that foster reflection, such as narrative medicine. Following this, educators need to propose appropriate strategies to address learners’ concerns so that these narrative approaches may potentially spur creative processes of thought and learning [64].

Since many participants expressed their struggles with engaging in writing narrative, such an act of writing did unfold with unexpected consequences. To make a shift in narratives and to achieve a state of inter-subjectivity appear to be facilitative for learners to grasp an opportunity for experiencing perspective-taking and transformation [65]. These findings are in line with Mezirow’s theory of transformative learning [66]. Attending to the stories narrated by participants can enhance their capacity of perspective-taking because they tend to ‘put themselves in patient’s shoes’. By ‘shifting to a narrative mode’, as one participant described, physicians can sensitize themselves to the surrounding stories with a heightened awareness [9]. Within the context of a small group discussion in narrative medicine, participants were able to shift roles between a teller and a listener and gain vicarious experiences of and insights to other peers’ stories. Brendel [67] proposed a narrative-driven transformative learning framework, linking the ideas of transformative learning and narrative medicine, to enhance understanding of where the learning is intended to take place, the intensity of reflection, and the subject matter considered through narratives. Despite the fact that the participants had been struggling to find ways to attain the desired goal of the course, the process of writing narratives turned out to be transformative because it introduced opportunities and engaged learners in shifting their roles in narratives, rediscovering the unnoticed details, and restructuring and making sense of their experiences [67].

The theme of seeking guidance arose from participant accounts, particularly in the context of fostering reflective capabilities in a course like narrative medicine. Back to the essence of reflection, reflection is inevitably initiated during a state of disorientation or confusion [18]. It is that moment when an individual needs guidance most. Previous literature on adopting narrative medicine to facilitate reflection has demonstrated guided reflection could lead to a fruitful outcome and gain learners’ appreciation [68]. Learners need guidance along their learning journey; that is, in different stages of the narrative medicine course, learners may require: (1) a communicative guidance to engage learners in a dialogue about narrative medicine; (2) a narrative writing guidance to support learners how to write about individual narrative assignments; and (3) a reflective guidance to facilitate or model reflection in small group discussion. In line with the concept of scaffolding, which typified adding supports to enhance learning and mastery of tasks [69], educators can strategically engage learners in reflection in a supportive and sustainable way by providing these forms of guidance to meet learner needs at different stages. Once the needed guidance is lacking, it may worsen the sense of uncertainty and deepen learner hesitancy to embark on a haphazard journey of reflection.

As participants expressed their eagerness to seek guidance, they also elaborated the impact of a trusting relationship with the assigned educator or group members on their lived experiences with narrative medicine. Regardless in the context of one-to-one or small group discussion, the literature on reflection has raised ethical concerns because reflection forces educators to create a space for learners to expose personal authentic emotions and vulnerabilities, conflict self-assessment with objective judgment, or challenge individual presumptions with societal needs [35]. Building a trusting relationship between the educator and learners takes time, space, and mutual endeavors. It is daunting for individuals who find themselves in unfamiliar groups in a narrative medicine context and, of course, even more challenging for them to share personal narratives. However, in order to develop reflective capabilities in narrative medicine, learners should be paired for a sustained period with a trusted educator who is able to facilitate the skills of empathetic listening, modeling personal reflection, and coaching learners through their transitions to become a doctor. This ideal relationship is analogous to the ‘pedagogical relation’ extrapolated by van Manen [70], which is ‘the concept of a caring human vitality that captures the normative and qualitative features of educational processes’ (p. 149). The intentional characteristics of the pedagogical relation enable educators to care for learners as they are or for what they may become. To maintain this relation, an educator needs to understand the present situation of learners and anticipate the moments when learners in fuller responsibility can progressively participate in the culture of medical profession. Hence, pedagogical relation is an intense life experience in which the relationship to an educator is the one which strongly fulfills and shapes learner being [70]. To this end, the pedagogue, like narrative medicine, should be ‘a supporter along the way…and creating the conditions and secure spaces for young people to play an active part in their own becoming’ (p. 162) [70].

While learning can be conceptualized as participation in social and cultural contexts [71, 72], our participants invariably experienced the influences of the learning environment on their learning and practice of reflection in narrative medicine. Many aspects in the learning environment, such as educator preparedness, learner motivation, deliverance of the narrative medicine course, and arrangement of small group discussion, contribute to the learning climate perceived by the learners [73]. These findings are consistent with the literature on factors contributing to construct a supportive learning environment in workplace learning [8, 74, 75]. Boud and Walker [15] argued that ‘context is perhaps the single most important influence on reflection and learning’ (p. 196) and asserted that educators need to ‘create a micro-context within which the kinds of reflection acceptable to learners and consistent with the values of learners and teachers can occur’ (p. 202) [15]. If educators intend to promote learner reflection in narrative medicine or other professional courses, it is fundamental to pay attention to and respect learners’ agendas which are shaped by their experiences, assumptions and practices of the larger context. From our findings that illustrate the essence of the phenomenon, as depicted in Fig. 1, educators can facilitate a smoother journey for learners by tackling the elements that may impede the construction of the micro-context in a narrative medicine course.

### Strengths of this study and limitations

By applying the principles of transcendental phenomenology, a clearer picture of participants’ lived experiences was disclosed through Moustakas’ systematic and rigorous approaches to data analysis. The essence of the phenomenon not only represents learners’ lived experiences, but also constitutes the meaning learners ascribe to their experiences with narrative medicine. We believe that these results are informative for educators who intend to adopt similar narrative approaches, or other genres of educational methods, strategically to foster reflection in medical learners in different contexts. Since clinical environment is the main arena for training reflective capabilities in medical education, the model we presented can help to explain the dimensions that educators need to consider upon designing and implementing curricula to facilitate learners to become reflective practitioners.

This study has several limitations. First, it was conducted in a single department at one tertiary medical center in Northern Taiwan. Although this context was unique to the present study and the results could be interpreted as lived experiences on a particular narrative medicine course, we suggest these findings are invaluable as fostering reflection has been regarded as beneficial for learning in different disciplines and can be informative for medical learners and educators in other contexts adopting similar narrative approaches. Second, the course consisted of one lecture, one narrative writing session and one small group discussion, and spanned over a three-month clerkship rotation in internal medicine. This short exposure may not allow a lot of lived experience to go on and lead to the desired outcomes. Using a systematic approach of phenomenological analysis, we believe the findings are informative for educators to develop a deeper understanding about the features of participant learning in narrative medicine. Future studies may consider following a cohort of learners with a longitudinal curricular design to demonstrate and evaluate the actual impacts of narrative medicine on learning reflection by learners. Third, the essence of any experience is never totally exhaustive [51]. The results can only reflect the experiences of the participants interviewed at a particular time and place. It is also unclear whether the participants recruited might have impact on the results since they might have shown more interest in or had benefited from the course. Even so, based on the rich data participants shared during the interviews and the application of Moustakas’ systematic phenomenological analysis, we attained a better understanding about the phenomenon and added new knowledge to current literature. Fourth, it is difficult to achieve the status of *epoche.* However, through this intentional process, the researchers were enabled to develop sensitivity to acknowledge and reduce the influence of preconceived thoughts, judgements, experiences and biases [55].. Nonetheless, given efforts from a diversity of team members with expertise in educational research and qualitative studies, the research team set aside personal biases and prepositions carefully through iterative discussion and critical reflection along the processes of conducting this research [53]. Finally, although an insider’s knowledge is advantageous in phenomenological design, the previous role of the primary researcher as an educator in the course may have limited participants’ disclosure. However, from participants’ candid responses and provision of rich data, the interest and appreciation we showed in their participation seemed to have encouraged them to share their perspectives freely and engage them in reflecting on their learning experiences in the narrative medicine course.

### Implications and future research directions

The results of this study may inform both learners and educators who will be involved in similar curricula or narrative-based activities and elucidate what the essence constitutes the journey they will undertake. With a clear delineation of the essence of the phenomenon, educators and institutions should pay attention to how to develop strategies to provide sufficient guidance, secure educator-learner and learner-learner relationships, prepare safe and supportive learning environments. In doing so, learner hesitancy can be alleviated from different angles so that they can be better facilitated to embark on a reflective journey. In particular, modeling reflection within the guidance of a trusted educator appears to be a powerful intervention to support reflective learning in medical learners. Future research questions may include: What specific professional development activities will provide medical educators with the required skills to facilitate reflection? How can educators create trusting pedagogical relations with learners in the context of narrative medicine or adopting similar narrative approaches? How can a learning environment provide supports and scaffolds to foster reflective learning?

## Conclusions

Through the adoption of a phenomenological design and Moustakas’ transcendental phenomenology approach, this study illuminated the lived experiences of medical learners regarding learning with narrative medicine and the meanings learners ascribed to the phenomenon. Learner hesitancy should be tackled with care by educators and supporting learners with strategies that address guidance, relationship, and learning environment. In so doing, medical learners can be facilitated to develop reflective capabilities for professional and personal growth.

## Data Availability

The data collected in this study are not publicly available due to confidentiality issues. The anonymized data will be shared by the corresponding author upon reasonable request.

## Appendices

### Appendix 1

Interview guide.

## 1. Tell me about your experience with the narrative medicine course

1. How was it organized?
2. What dimensions, incidents or people connected with the experience stand out for you? Please give me an example or your story about it.
3. How did the experience affect you?

## 2. Recount your experience with the narrative medicine course

1. When have you experienced reflection in narrative medicine?

Specify by recounting what took place in an actual learning session.

Probe: How did you perceive reflection’s role in narrative medicine?

Probe: How did you learn or practice reflection in narrative medicine?

Please give me an example or your story about it.

(2) Which contexts or situations have affected your experiences of reflection in narrative medicine?

Probe: Have you ever experienced any obstacles to learn reflection in narrative medicine? If yes, please describe the obstacle, when and where, and how you dealt with it.

Probe: What supports do you think you need to learn reflection in the course?

Please describe it.

### Appendix 2

Summary of major processes of Moustakas’ transcendental phenomenological analysis (Moustakas, 1994).

**Table:**

Process	Description
*Epoche (Bracketing)*	At the beginning of the study, researchers set aside prejudgments, biases, and preconceived ideas about the phenomenon of the study interest based on prior experiences. This process allows researchers to enter anew into consciousness, and prepare a receptive and open-minded attitude to focus on the views reported by the participants.
Phenomenological reduction (textural description)	Significant statements pertaining to the experiences of the participants are identified by researchers through iterative acts of close reading. This compilation grounds the *horizons* to acknowledge the textural meanings and invariant constituents of the phenomenon (*horizonalization*). The researchers then cluster these statements into meaning units or themes, and organize the horizons and themes into a coherent *textural description* of the phenomenon to illuminate the “what” of the experience.
Imaginative variation (structural description)	Researchers seek to possible meanings through utilization of imagination, approaching the phenomenon from different perspectives or varying the frames of reference. This process aims to present *structural descriptions* of an experience and the factors contributing to what is being experienced by the participants; that is, the delineation of the “how” for what is being experienced.
Synthesis (composite description of the phenomenon)	Researchers intuitively integrate the fundamental textural and structural descriptions into a unified, composite statement, explicating the essence and meaning of the experience of the phenomenon as a whole.

### Appendix 3

Selected examples of significant statements.

**Table:**

1. When we initially heard that narrative medicine was similar to other courses of medical humanities, our first response was “Again, they are attempting to give us some high-sounding knowledge or distant, irrelevant concepts”. Frankly speaking, I almost run out of interest to learn. (P2, F, MS5) 2. Because I have not joined the group discussion, I am doubtful about the proclaimed benefits of narrative medicine.(P4, M, MS6) 3. In the introductory session of narrative medicine, there were several clinical scenarios and terms related to narrative competence or medical ethics. I contended that these were high sounding clichés because of lacking similar experiences. … Those case scenarios were not authentic and we perceived no relevance to ourselves. (P7, F, MS5) 4. Writing a composition (to learn how to reflect) is strange to me. I am not persuaded that learners could gain reflective capabilities by merely writing up a good story.(P10, M, MS6) 5. I don’t think just taking these kind of courses will be helpful to improve our communication skills or build a good doctor-patient relationship. (P6, F, R1)	
6. To me, this is a kind of shift. It is this shift that makes you change to another mode, a sensitive and humanistic mode. (P1, F, MS7) 7. After you did the writing work, you tried to persuade yourself that you had cared for the patient… you thought and gradually narrated the patient’s story and came to a realization: there were so many details you could pay attention to. This thoughtful feeling could hardly be achieved before I put the story into words. (P4, M, MS6) 8. If I was the patient’s mother, neither a physician nor a medical student, and I happened to know that this group of health professionals knew more truth (about my son’s illness) than me, I would be extremely upset…, and even reluctant to accept my role as the patient’s mother. (P3, F, MS5) 9. It is only when you write the narrative that you would particularly want to highlight some points. It (narrative medicine) reminds you of paying attention to what happened in the wards and facilitates your thinking about what reasons are for the events you encountered with patients. (P10, M, MS6) 10. I may not have the answer to this issue if I just think over it again. However, writing it down provided me with an opportunity to investigate the details. In comparison to that it took only 1 sec when the patient dropped his tears, the time could be amplified to 10 sec when you narrate the story or review the experience one more time. (P15, F, MS6)	
11. I gained more feedback on emotional dimension. I shared my feelings and found out that echoed among most of the colleagues because we had similar experiences and feelings…. Though I did not get the answer I expected but the feedback from colleagues resonated within my heart. (P7, F, MS5) 12. When I reflected on my writing, instead of criticizing the phenomenon as a doctor, I learned that I should put myself in patient’s shoes to think about the ailments he had, and the situation that his family encountered, and what kinds of help I could offer as one of his healthcare professionals. (P10, M, MS6) 13. For me, narrative medicine inspired me to think about the roles of being a physician, and what kinds of a physician I want to be. (P10, M, MS6) 14. In my narrative, I detected that the patient was sad and tried to accompany him in those days. After sharing the story in my group, I turned to think about how to collaborate with patient’s families to keep the patient calm in confrontation with his ailment. The strategies may not merely depend on the words I choose in communication but, more importantly, stem from the attitude I adopt or the atmosphere I create through my practice. (P15, F, MS6) 15. As I described in my narrative, I was afraid of being incapable of dealing with the patient’s emotions at the clinic. I came up with a solution in my writing which was: maybe at that time I could have brought the patient to another quiet room and let the rushing pace slow down. (P2, F, MS5)	
16. Before writing our narratives, I guess, most of the students did not know how and what to write. I thought we needed some hints and guidance. The grading also impeded the processes of writing. We needed to be informed that we could choose freely what we want to write and the word counts would not impact the grading. (P7, F, MS5) 17. I felt that we were given a haphazard journey. We did not receive the needed guidance…. I did not know how to write a narrative or how to find a starting point. Frankly speaking, I had doubted about this for a while. (P1, F, MS7) 18. If the instructor want to achieve the goals of this course, it would be helpful for him to explain more details about how to write narrative assignments. (P7, F, MS5) 19. After the introductory class, I spent a while to look over the posted narratives on the website in order to understand some details about how to prepare for this assignment.(P3, F, MS5) 20. The educator plays a critical role. If he disregard this course or lack of facilitation skills to lead a small group and guide heated discussion, the class would turn out to be a formality.(P16, F, MS6)	
21. As a matter of fact, we were very anxious in a small group setting because the educator asked everyone to comment on another member’s narrative. For any reason, you would try not to give negative feedback. Hence, most comments fell into the similar (superficial) category. I did not expect too much for the discussion. (P1, F, MS7) 22. In fact, I barely knew the educator, the facilitator in our small group. I had never seen him in the hospital … Apparently, it was difficult for me to discuss about anything in that situation since I did not know him. (P2, F, MS5) 23. The acquaintance level between the members is very important. There were some members from Chinese Medicine in our group and they did not express themselves very well or tended to be silent in discussion because they (might thought they)were not part of the core. The level of familiarity would make the discussion more heated. (P4, M, MS6) 24. There is someone in the group who you are not acquainted with or who you will shy away to share your narrative. This issue (familiarity) may embarrass people. (P15, F, MS6) 25. As for the group members, we did not know each other very well ….just nodding acquaintance. They were not the classmates you would like to tell them the truth from the bottom of your heart.(P3, F, MS5) 26. If you dislike someone in your group, you will wonder why you should share your narrative and reflections to him. (P17, F, MS7)	
27. As a matter of fact, we were very anxious in a small group setting because the educator asked everyone to comment on another member’s narrative. For any reason, you would try not to give negative feedback. Hence, most comments fell into the similar category and were superficial. (P1, F, MS7) 28. The educator was rather open and would not force everyone to speak in the discussion. He created a climate for us to express our opinions voluntarily and not feel pressured to speak something in discussion. (P4, M, MS6) 29. Our educator invited us to a restaurant and we started group discussion after having our dinner. It was quite a relaxed atmosphere. He (the educator) asked us to read the narratives in our own way… The voices and tones you read the narrative expressed your own feelings. Such a climate enabled us to share… especially when someone sparked the flame with sharing personal experiences, another one would follow … The educator did not set up a time limit for each sharing. (P7, F, MS5) 30. In our group, the educator seemed unsure about what was this course for. He listened to everyone read personal narratives and that’s all. It was not effective anyway. Just having everyone read his or her narratives and that was quite an embarrassing situation…. Everyone presumed it was a formality and just finished reading as quickly as possible. It was meaningless. (P20, F, MS5) 31. The environment counts (for sharing our narratives). Some group held the discussion at a restaurant “The Rose Garden”, which was conducive for thinking and sharing. In contrast, our group members were crowded in a small meeting room. It was a tight squeeze and I just wanted to leave as soon as possible. (P12, F, MS6)	
